# Investigation of Allergic Sensitizations in Children With Allergic Rhinitis and/or Asthma

**DOI:** 10.3389/fped.2022.842293

**Published:** 2022-03-15

**Authors:** Lingman Dai, Jinling Liu, Qi Zhao, Mengyao Li, Yunlian Zhou, Zhimin Chen, Yuanyuan Zhang

**Affiliations:** ^1^Department of Pulmonology, Children's Hospital, Zhejiang University School of Medicine, National Clinical Research Center for Child Health, Hangzhou, China; ^2^Hangzhou Bay Hospital, Ningbo, China

**Keywords:** allergic rhinitis, children, skin prick tests, asthma, aeroallergens

## Abstract

**Objective:**

Allergic rhinitis (AR) and asthma are becoming one of the most prevalent diseases in children. Identifying sensitization to aeroallergens is seemed to be valuable for diagnosing allergic disease and guiding its treatment. This study aimed to analyze the profiles of sensitization to aeroallergen in children with AR and/or asthma by skin prick test (SPT) and explore the differences of sensitization between different kinds of allergic diseases, different sexes, and different ages.

**Methods:**

A total of 230 children with AR and/or asthma who were hospitalized in our hospital from June 2017 to September 2019 were eligible in this retrospective study. All patients completed standardized questionnaires and SPT. Based on the sex, age, or classification of allergic disease, the sensitizations to 13 aeroallergens were compared.

**Results:**

Of the 230 patients, 67.4% of enrolled were positive for SPT; the top 5 allergens were *Dermatophagoides pteronyssinus* (*Der.p*) (59.3%), *Dermatophagoides farina* (*Der.f* ) (58.7%), *Blomia tropicalis* (*Blot*.) (40.3%), dog hair (36.1%), and *Blattella germanica* (20.4%). More than 90% of patients were sensitized to two or more allergens. As to the effect of age on aeroallergens, we found that the sensitizations of *Blot*., dog fur, and multiple sensitizations (≥5 allergens) were more common in adolescence (*P* < 0.01, *P* < 0.05). Regarding sex, we found that the positive rate of SPT and the percentages of double-allergen sensitizations in boys were much higher than girls (*P* < 0.01, *P* < 0.01), and the positive rate to *Der.p, Der.f* , and ragweed were also significantly higher in boys (*P* < 0.01, *P* < 0.05, and *P* < 0.05, respectively). Furthermore, we found that asthma–rhinitis multimorbidity increased the incidences of sensitizations; patients with AR and asthma had significantly higher positive rates to *Der.p* and *Der.f* when compared with the AR or asthma group (*P* < 0.05, *P* < 0.05).

**Conclusion:**

Allergic sensitizations were common in children with AR and/or asthma; sex, age, and asthma–rhinitis multimorbidity might affect the prevalence of sensitizations to aeroallergens.

## Background

Allergic diseases are a collection of disorders caused by the hypersensitivity of the immune system to allergens, which could dramatically impair the quality of life of patients and sometimes lead to life-threatening situations, thus, causing a huge socioeconomic burden (1). Allergic rhinitis (AR) and asthma are the most prevalent allergic diseases in children, and the prevalence of AR and asthma in children have been estimated to be ~2–25 and 3–38%, respectively (2, 3). In addition, a World Health Organization report revealed the close link between AR and asthma, which showed that ~30% of patients with rhinitis develop asthma and 74–81% of patients with asthma have rhinitis (4–6).

Exposure to sensitized allergens is a critical trigger factor in the development of AR and asthma, which may exacerbate the symptoms of these diseases by promoting airway inflammation and hyperresponsiveness (7, 8). Skin prick test (SPT) is one of the essential methods widely applied for identifying sensitization to aeroallergens, which can provide valuable evidence for the diagnosis and personal specific immune therapy of AR and asthma (9). Previous studies focused on the pattern of sensitization to allergen among adult patients and the prevalent differences of the region and season (8, 10, 11).

So, in this study, we retrospectively analyzed the profiles of sensitization to aeroallergen in children with AR and/or asthma who underwent SPT in our hospital between June 1, 2017, and September 31, 2019, and explored the differences of sensitization between different kinds of allergic diseases, different sexes, and different ages.

## Methods

### Participants

In our single-center retrospective study, we collected the clinical data of outpatients who completed standardized questionnaires and SPTs from June 2017 to September 2019 in Children's Hospital, Zhejiang University School of Medicine. The inclusion criteria for enrolling patients were as follows: (1) The patients were diagnosed with asthma alone, rhinitis alone, or asthma accompanied with rhinitis. The diagnoses of AR and asthma were based on the Allergic Rhinitis and its Impact on Asthma guidelines (rhinitis) (12) and Global Initiative for Asthma criteria (asthma) (13), respectively; (2) patients aged between 5 and 18 years. The exclusion criteria were as follows: (1) had received antihistamines, sodium chromatin, leukotriene receptor antagonist, or systemic glucocorticoids within 7 days; (2) had anaphylaxis attack within 1 month or had a history of severe allergic reactions; (3) had an acute asthma attack or forced expiratory volume in 1 s/forced vital capacity <70% predicted; (4) local skin had severe dermatitis preventing execution and interpretation of SPTs; (5) had accepted specific immunotherapy before; (6) had incomplete medical records.

### Data Collection

The patients and/or their guardians completed the questionnaires, which were used in the survey, which were adopted from the Chinese translated version of the questionnaire from International Study of Asthma and Allergies in Childhood phase II, and modifications were made according to the real situation of China (14). Several questions in the questionnaire, including basic information (name, age, and sex), family history of allergic diseases, symptoms of rhinitis, wheezing, or coughing, eczema, and burning or itchy eyes, etc. were asked by the physicians or research nurses face to face. The ethics committee of the Children's Hospital, Zhejiang University School of Medicine (2018-IRB-07), approved the study.

### Allergens Used in Skin Prick Test

Patients were subjected to SPT for 13 aeroallergens extracts, including *Dermatophagoides pteronyssinus* (*Der.p*)*, Dermatophagoides farina* (*Der.f* )*, Blomia tropicalis* (*Blot*.), cat fur, dog hair, *Blattella germanica*, American cockroach, white birch, ragweed, mugwort, mixed grass, *Alternaria*, and *Aspergillus*. Aeroallergen extracts and control solutions were obtained from ALK (Horsholm, Denmark). Histamine (10 mg/ml) and 0.9% sterile saline were used as positive and negative controls, respectively.

### Skin Prick Tests

Pricking was performed on the volar side of the forearm, and the adjacent pricking interval area was more than 2 cm. We sterilized the forearms of patients with a 75% alcohol swab, a needle was inserted vertically into the skin by a professional allergy nurse, and pricks were administered with consistent intensity and depth. After 15 min, results were measured as the mean of the longest diameter and the length of the perpendicular line through its middle. A wheal size of more than 3 mm was considered a positive result after subtraction of the negative control. The result was also expressed as skin index (SI = mean size of allergen weal/mean size of histamine wheal). The diagnostic criteria were as follows: level 1, SI = 0, “negative”; level 2, 0 < SI < 0.5, “+”; level 3, 0.5 ≤ SI < 1.0, “++”; level 4, 1.0 ≤ SI < 2.0, “+++”; level 5, SI ≤ 2.0, “++++” (8, 10).

### Statistics

SPSS25.0 statistical software (IBM, Armonk, NY, USA) was used for data analysis. Non-normally distributed continuous variables were presented as the median (interquartile range), and groups were compared by Mann–Whitney *U*-test. Categorical data were shown as number (%) and analyzed by Chi-squared tests. Spearman rank-correlation coefficients were used to describe the association between different variables and SPT positive rate. Statistical significance was defined as *P* < 0.05.

## Results

### Allergic Sensitizations in All Patients

**Figure 1:**
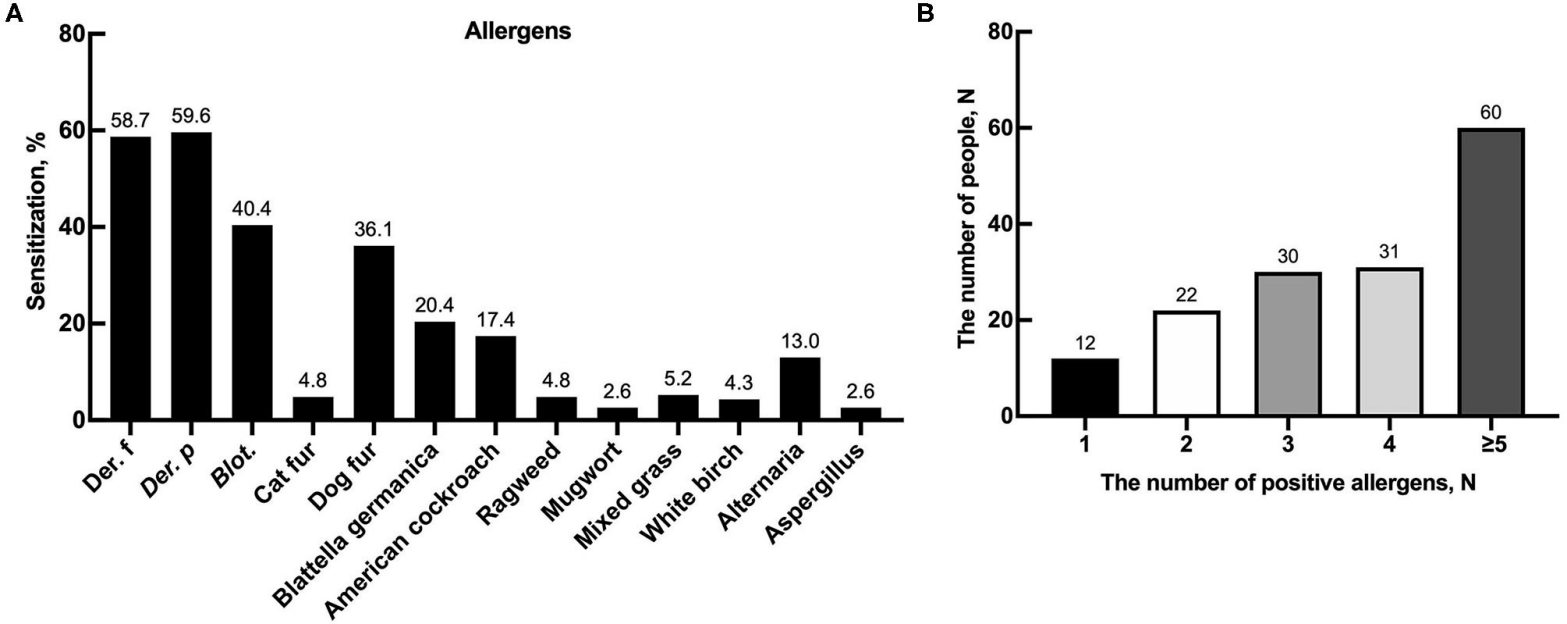
Allergic sensitizations in patients. **(A)** Sensitization to 13 aeroallergens based on SPTs in children with AR and/or asthma. **(B)** The number of people in different number of positive allergens in all person.

A total of 230 patients with AR and/or asthma from June 2017 to September 2019 were eligible in this study, including 182 cases (109 boys, 73 girls) in childhood (5–10 years) and 48 cases (34 boys, 14 girls) in adolescence (11–18 years). The median age of patients was 8.8 (7.4–10.6) years, with a female-to-male ratio of 0.61. Of the 230 patients, 155 (67.4%) had at least one positive skin reaction, and 91 patients (39.6%) had a family history of allergic disease. The overall prevalence of positive skin responses is shown in Figure 1A. The SPT sensitization rates in descending order were *Der.p* (59.3%), *Der.f* (58.7%), *Blot*. (40.3%), dog hair (36.1%), *B. germanica* (20.4%), American cockroach (17.4%), *Alternaria* (13.0%), mixed grass (5.2%), cat fur (4.8%), ragweed (4.8%), white birch (4.3%), mugwort (2.6%), and *Aspergillus* (2.6%). Among the 155 patients with positive SPT reaction, 12 (7.7%) cases were sensitized to one allergen, 22 (14.2%) cases were sensitized to 2 allergens, 30 (19.4%) cases were sensitized to 3 allergens, 31 (20.0%) cases were sensitized to 4 allergens, and 60 (38.7%) cases were sensitized to 5 or more allergens (Figure 1B).

### Differences of Allergic Sensitizations Among Different Ages

Based on age, patients were divided into childhood and adolescence. As can be seen in Table 1, we found that there were no significant differences in sex distribution and positive rate of SPT between these two groups (*P* > 0.05). However, sensitivities against *Blot* (*P* < 0.01) and dog hair (*P* < 0.01) were significantly increased in adolescents than in childhood, whereas sensitivities against other 11 allergens were similar (*P* > 0.05). Meanwhile, we found a significantly greater number of childhood were sensitized to three allergens than adolescents (*P* <0.01); In contrast, the percentage of more than five allergen-sensitizations in adolescents was higher than that in childhood (*P* < 0.05) (shown in Figure 2A).

**Table 1:** Comparison of sensitized allergens among different ages.

	**Childhood (*n* = 182)**	**Adolescent (*n* = 48)**	***X* ^2^**	***P***
**Sex**				
Boy	109 (59.9%)	34 (70.8%)	1.934	0.164
Girl	73 (40.1%)	14 (29.2%)		
SPT positive rate	122 (67.0%)	33 (68.8%)	0.512	0.821
**Sensitized allergens**				
*Der.p*	104 (57.1%)	33 (68.8%)	2.125	0.145
*Der.f*	103 (56.6%)	32 (66.7%)	1.590	0.207
*Blot*.	65 (35.7%)	28 (58.3%)	8.068	0.005^#^
Cat fur	7 (3.8%)	4 (8.3%)	1.679	0.195
Dog hair	56 (30.8%)	27 (56.3%)	10.692	0.001^#^
*Blattella germanica*	33 (18.1%)	14 (29.2%)	2.845	0.092
*American cockroach*	29 (15.9%)	11 (22.9%)	1.289	0.256
Ragweed	9 (4.9%)	2 (4.2%)	0.051	0.822
Mugwort	5 (2.7%)	1 (2.1%)	0.066	0.797
Mixed grass	11 (6.0%)	1 (2.1%)	1.205	0.272
White birch	9 (4.9%)	1 (2.1%)	0.748	0.387
*Alternaria*	26 (14.3%)	4 (8.3%)	1.187	0.276
*Aspergillus*	5 (2.7%)	1 (2.1%)	0.066	0.797

*Der.p, Dermatophagoides pteronyssinus; Der.f, Dermatophagoides farinae; Blot., Blomia tropicalis*.

*Data are presented as number (percentage)*.

# *P < 0.01*.

**Figure 2:**
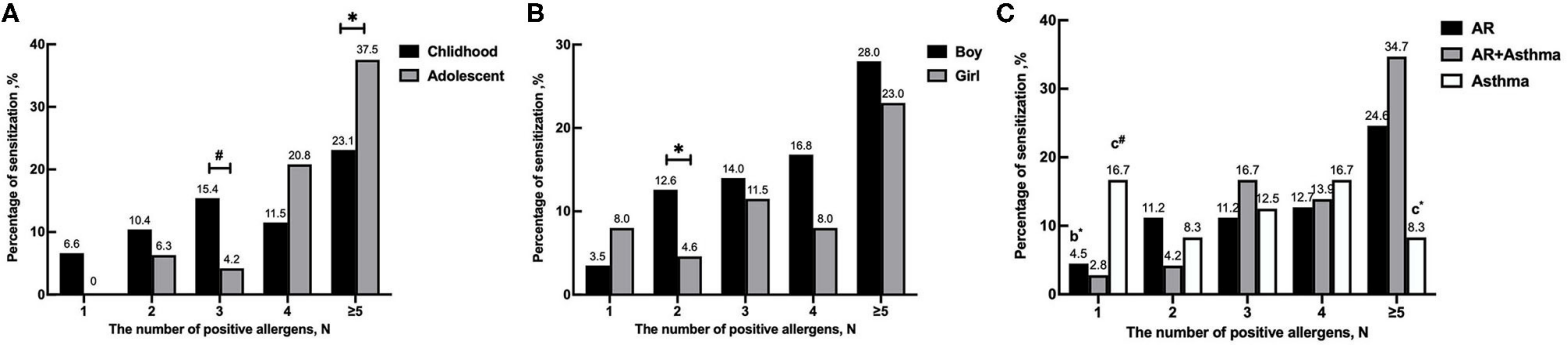
Sensitization to different number of positive allergens in different ages, genders and disease groups. **(A)** The number of positive allergens in different ages. **(B)** The number of positive allergens in different genders. **(C)** The number of positive allergens in different diseases. *P < 0.05, ^#^*P* < 0.01. ^b^Compared between AR group and asthma group. ^c^Compared between AR combined with asthma group and asthma group.

### Differences in Allergic Sensitizations Among Different Genders

According to a different sex, differences in allergic sensitizations were compared. As shown in Table 2 and Figure 2B, we found that the positive rate of SPT in boys was much higher than in girls (74.8 vs. 52.9%, *P* < 0.01), and the percentages of double-allergen sensitizations in boys were also higher than in girls (*P* < 0.01), which showed significant differences (*P* < 0.01). Furthermore, we found that boys also had a significantly higher positive rate to *Der.p, Der.f* , and ragweed than girls (*P* < 0.01, *P* < 0.05, and *P* < 0.05, respectively). However, there were no statistically significant differences in sensitizations to the other 10 allergens (*P* > 0.05).

**Table 2:** Comparison of sensitized allergens between different genders.

	**Boy (*n* = 143)**	**Girl (*n* = 87)**	**Z/X^**2**^**	***P***
Age	8.49 (5.6–17.1)	8.48 (5.2–16.2)	0.474	0.635
SPT positive rate	107 (74.8%)	46 (52.9%)	9.507	0.002^#^
**Sensitized allergens**
*Der.p*	96 (67.1%)	41 (47.1%)	8.989	0.003^#^
*Der.f*	93 (65.0%)	42 (48.3%)	6.267	0.012^*^
*Blot*.	63 (44.1%)	30 (34.5%)	2.058	0.151
Cat fur	6 (4.2%)	5 (5.7%)	0.286	0.593
Dog hair	57 (39.9%)	26 (29.9%)	2.334	0.127
*Blattella germanica*	33 (23.1%)	14 (16.1%)	1.623	0.203
*American cockroach*	27 (18.9%)	13 (14.9%)	0.584	0.445
Ragweed	10 (7.0%)	1 (1.1%)	4.056	0.044^*^
Mugwort	5 (3.5%)	1 (1.1%)	1.173	0.279
Mixed grass	9 (6.3%)	3 (3.4%)	0.886	0.347
White birch	9 (6.3%)	1 (1.1%)	3.442	0.064
*Alternaria*	22 (15.4%)	8 (9.2%)	1.827	0.177
*Aspergillus*	2 (1.4%)	4 (4.6%)	2.179	0.140

*Der.p, Dermatophagoides pteronyssinus; Der.f, Dermatophagoides farinae; Blot., Blomia tropicalis*.

*Data are presented as number (percentage)*.

* *P <0.05*,

# *P < 0.01*.

### Differences of Allergic Sensitizations Among Different Diseases

According to the disease, patients were divided into three groups: the AR group (*n* = 134), the asthma group (*n* = 24), and the AR and asthma group (*n* = 72). As shown in Table 3, no differences were found in sex distribution and age (*P* > 0.05). The positive rate of SPT in children with AR and asthma was higher than that in the other two groups (76.4% in the AR and asthma group, 64.2% in the AR group, and 58.3% in the asthma group), but no statistically significant difference was found among these groups (*P* > 0.05). Interestingly, we found that the prevalence of sensitizations to *Der.p* and *Der.f* was significantly different among these three groups (*P* < 0.05, *P* < 0.05), but there were no statistically significant differences in the prevalence of sensitizations to other 11 allergens (*P* > 0.05). We also found that patients with AR and asthma had significantly higher positive rates to *Der.p* and *Der.f* when compared with the other two groups (*P* < 0.05, *P* < 0.05). In addition, the percentage of single allergen-sensitization in children with AR and asthma was lower than that in the other groups (2.8% in the AR and asthma group, 4.5% in the AR group, and 16.7% in the asthma group; *P* < 0.05, *P* < 0.01, respectively), whereas the percentages of more than five allergen-sensitizations were higher than those in the asthma group (*P* < 0.05) (Figure 2C).

**Table 3:** Comparison of sensitized allergens among different diseases.

	**AR**	**AR + asthma**	**Asthma**	**Z/X^**2**^**	***P***
	**(*****n*** **=** **134)**	**(*****n*** **=** **72)**	**(*****n*** **=** **24)**		
**Sex**					
Boy	83 (61.9%)	45 (62.5%)	15 (62.5%)	0.007	0.996
Girl	51 (38.1%)	27 (37.5%)	9 (37.5%)		
Age	8.49 (5.3–17.1)	8.71 (5.3–17.1)	7.6 (5.8–13.3)	2.789	0.248
SPT positive rate	86 (64.2%)	55 (76.4%)	14 (58.3%)	4.178	0.124
**Sensitized allergens**					
*Der.p*	75 (56.0%)	51 (70.8%)^a^^*^^,^ ^b^^*^	11 (45.8%)	6.394	0.041
*Der.f*	72 (53.7%)	52 (72.2%)^a^^*^^,^ ^b^^*^	11 (45.8%)	8.434	0.015
*Blot*.	51 (38.1%)	36 (50.0%)	6 (25.0%)	5.423	0.066
Cat fur	7 (5.2%)	4 (5.6%)	0 (0.0%)	1.375	0.507
Dog hair	44 (32.8%)	32 (44.4%)	7 (29.2%)	3.293	0.193
*Blattella germanica*	29 (21.6%)	16 (22.2%)	2 (8.3%)	2.423	0.298
*American cockroach*	23 (17.2%)	15 (20.8%)	2 (8.3%)	1.969	0.374
Ragweed	5 (3.7%)	6 (8.3%)	0 (0.0%)	3.524	0.172
Mugwort	4 (3.0%)	2 (2.8%)	0 (0.0%)	0.726	0.696
Mixed grass	7 (5.2%)	5 (6.9%)	0 (0.0%)	1.755	0.416
White birch	5 (3.7%)	5 (6.9%)	0 (0.0%)	2.381	0.304
*Alternaria*	14 (10.4%)	14 (19.4%)	2 (8.3%)	3.866	0.145
*Aspergillus*	3 (2.2%)	2 (2.8%)	1 (4.2%)	0.310	0.857

*Der.p, Dermatophagoides pteronyssinus; Der.f, Dermatophagoides farinae; Blot., Blomia tropicalis*.

*Data are presented as number (percentage)*.

* *P < 0.05*.

a *Compared between AR group and AR combined with asthma group*.

b *Compared between AR combined with asthma group and asthma group*.

## Discussion

AR and asthma are two of the most common allergic diseases in children, which may often coexist. AR and asthma sometimes could be triggered when exposed to sensitized allergens (4), so SPT had been generally performed for clinical implication. Up to now, there have been several studies focused on the sensitization to aeroallergens measured by SPT. However, there is little published literature focusing on the profiles of sensitization to aeroallergen in children. Our study described the distribution of sensitizations to allergens and characteristics in children with AR and/or asthma from Zhejiang Province. Meanwhile, our research analyzed the differences of allergic sensitization between different kinds of allergic diseases, different sexes, and different ages.

Our SPT results indicated that the most common allergen in children from Zhejiang Province is *Der.p* and followed by descending order, *Der.f, Blot*., dog hair, *B. germanica*, American cockroach, *Alternaria*, mixed grass, cat fur, ragweed, white birch, mugwort, and *Aspergillus*, which were similar with another study (10). A Qatar study showed that the top 5 common aeroallergens were *Der.p* (38.1%), *Der.f* (29.0%), cat (22.6%), *Alternaria* (18.8%), and American cockroach (18.4%) in children with AR combined with asthma (15). The positive rates of the top 5 common aeroallergens differed, which was considerable heterogeneity of sensitization schema between different regions. Furthermore, we found that multiple sensitizations are common in our study, and more than 90% of patients were sensitized to two or more allergens, which were confirmed in another study (10).

Our study studied the effects of age and sex on the sensitization to aeroallergens. Our results showed that the sensitizations of *Blot* and dog fur were more common in adolescence than those in childhood, which were similar to other studies. Moral et al. (16) showed that the mean age of patients with asthma and/or AR sensitized to mites is 9.5 and 9.6 years to dog furs. Li et al. (10) found that a significantly higher percentage of patients in adolescence was sensitized to a dog. These results may be due to the fact that pet ownership is more common among them. Meanwhile, we found that multiple sensitizations (≥5 allergens) were also more common in adolescence. This result might be associated with a remarkable correlation between polysensitization to sensitization clusters with increasing age (17). As to the sex-related difference, we found that the positive rate of allergens and the percentages of double-allergen sensitizations in boys were substantially higher in the total population, and the sensitizations of *Der.f, Der.p*, and ragweed in boys were higher than those in girls. The reason for sex differentiation may be related to hormone levels (18, 19). However, further studies should be conducted to unveil the precise mechanisms related to the differences in age and sex.

It is well-known that exposure to sensitized allergens is a critical trigger factor in allergic disease and may influence its progress. So, in this study, we tried to investigate the differences of sensitization to aeroallergens between different allergic diseases in children. Interestingly, we found that the sensitizations to *Der.p* and *Der.f* were obviously higher, and the multiple sensitizations were much more common in the AR and asthma group than those in the other two groups, which were similar in other research in adults (10, 20). All these results indicated that the mechanisms involved in asthma–rhinitis multimorbidity might be partly different or enhanced than those involved in asthma or rhinitis.

There were several limitations in our study: First, healthy children were not enrolled in our study as blank control, which might lead to some deviation in the prevalence rate of each allergen in different populations. Second, the climate and geographical characteristics were not involved in our study, which might reduce its interest to readers. Third, we had not evaluated the relationship between the sensitization of aeroallergens and the severity of the allergic disease. Fourth, we had not also evaluated the relationship between SPT data and serum total immunoglobulin E or specific immunoglobulin E. So, further research should be performed to increase its potential impact on the general audience.

## Conclusion

In summary, our study showed that 67.4% of children with AR and/or asthma had sensitized to aeroallergens detected by SPT, more than 90% of patients were sensitized to two or more allergens, and more than half of patients were sensitized to *Der.p* and *Der.f* . The prevalence of sensitizations to allergens differed with sex or age, and asthma–rhinitis multimorbidity might increase the incidence of sensitizations. For such patients with allergic sensitization, more attention should be paid to clinical work, and corresponding management should be developed accordingly.

## Data Availability Statement

The original contributions presented in the study are included in the article/supplementary material, further inquiries can be directed to the corresponding author/s.

## Ethics Statement

The studies involving human participants were reviewed and approved by the Ethics Committee of the Children's Hospital, Zhejiang University School of Medicine (2018-IRB-07). Written informed consent to participate in this study was provided by the participants' legal guardian/next of kin.

## Author Contributions

LD and YZ designed the study and wrote the initial manuscript. JL and ZC designed the questionnaire. JL and YZ collected the data. QZ and ML analyzed the data. All authors read and approved the final manuscript.

## Funding

This work was partially supported by grants from the National Natural Science Foundation (81871264) and the Foundation for the Top-Notch Youth Talent Cultivation Project of Independent Design Project of the National Clinical Research Center for Child Health (Q21C0001) and Key Research and Development Program of Zhejiang Province (2020C03062).

## Conflict of Interest

The authors declare that the research was conducted in the absence of any commercial or financial relationships that could be construed as a potential conflict of interest.

## Publisher's Note

All claims expressed in this article are solely those of the authors and do not necessarily represent those of their affiliated organizations, or those of the publisher, the editors and the reviewers. Any product that may be evaluated in this article, or claim that may be made by its manufacturer, is not guaranteed or endorsed by the publisher.

## References

[1] Yao Y Chen CL Yu D Liu Z. Roles of follicular helper and regulatory T cells in allergic diseases and allergen immunotherapy. Allergy. (2021) 76:456–70. 10.1111/all.14639 33098663

[2] Brozek JL Bousquet J Agache I Agarwal A Bachert C Bosnic-Anticevich S. Allergic Rhinitis and its Impact on Asthma (ARIA) guidelines-2016 revision. J Allergy Clin Immunol. (2017) 140:950–8. 10.1016/j.jaci.2017.03.050 28602936

[3] Aaron SD Boulet LP Reddel HK Gershon AS. Underdiagnosis and overdiagnosis of asthma. Am J Respir Crit Care Med. (2018) 198:1012–20. 10.1164/rccm.201804-0682CI 29756989

[4] Testa D DI Bari M Nunziata M Cristofaro G Massaro G Marcuccio G. Allergic rhinitis and asthma assessment of risk factors in pediatric patients: a systematic review. Int J Pediatr Otorhinolaryngol. (2020) 129:109759. 10.1016/j.ijporl.2019.109759 31734564

[5] Yang L Fu J Zhou Y. Research progress in atopic march. Front Immunol. (2020) 11:1907. 10.3389/fimmu.2020.01907 32973790 PMC7482645

[6] Shaaban R Zureik M Soussan D Neukirch C Heinrich J Sunyer J. Rhinitis and onset of asthma: a longitudinal population-based study. Lancet. (2008) 372:1049–57. 10.1016/S0140-6736(08)61446-4 18805333

[7] Burrows B Martinez FD Halonen M Barbee RA Cline MG. Association of asthma with serum IgE levels and skin-test reactivity to allergens. N Engl J Med. (1989) 320:271–7. 10.1056/NEJM198902023200502 2911321

[8] Li J Huang Y Lin X Zhao D Tan G Wu J. Factors associated with allergen sensitizations in patients with asthma and/or rhinitis in China. Am J Rhinol Allergy. (2012) 26:85–91. 10.2500/ajra.2012.26.3751 22369791

[9] Yan YR Xu YH Zheng Q Guo YS. The prevalence and sex difference of allergen sensitization among adult patients with allergic diseases in Shanghai, China. Asian Pac J Allergy Immunol. (2019) 37:147–53. 10.12932/AP-150118-0241 29981565

[10] Li J Sun B Huang Y Lin X Zhao D Tan G. A multicentre study assessing the prevalence of sensitizations in patients with asthma and/or rhinitis in China. Allergy. (2009) 64:1083–92. 10.1111/j.1398-9995.2009.01967.x 19210346

[11] Dey D Mondal P Laha A Sarkar T Moitra S Bhattacharyya S. Sensitization to common aeroallergens in the atopic population of West Bengal, India: an investigation by skin prick test. Int Arch Allergy Immunol. (2019) 178:60–5. 10.1159/000492584 30257248

[12] Bousquet J Khaltaev N Cruz AA Denburg J Fokkens WJ Togias A. Allergic Rhinitis and its Impact on Asthma (ARIA) 2008 update (in collaboration with the World Health Organization, GA(2)LEN and AllerGen). Allergy. (2008) 63:8–160. 10.1111/j.1398-9995.2007.01620.x 18331513

[13] Global Initiative for Asthma. Global Strategy for Asthma Management Prevention. Available online at: http://www.globalasthmareport.org/Global Asthma Report (2017). pdf

[14] International Study of Asthma and Allergies in Childhood. Available online at http://isaac.auckland.ac.nz/phases/phasetwo/phasetwomodules.pdf; accessed (2011).

[15] Zahraldin K Chandra P Tuffaha A Ehlayel M. Sensitization to common allergens among children with asthma and allergic rhinitis in Qatar. J Asthma Allergy. (2021) 14:287–92. 10.2147/JAA.S295228 33824594 PMC8018446

[16] Moral L Roig M Garde J Alós A Toral T Fuentes MJ. Allergen sensitization in children with asthma and rhinitis: marked variations related to age and microgeographical factors. Allergol Immunopathol. (2008) 36:128–33. 10.1016/S0301-0546(08)72536-9 18680699

[17] Howard R Belgrave D Papastamoulis P Simpson A Rattray M Custovic A. Evolution of IgE responses to multiple allergen components throughout childhood. J Allergy Clin Immunol. (2018) 142:1322–30. 10.1016/j.jaci.2017.11.064 29428391 PMC6170973

[18] Rahimian N Aghajanpour M Jouybari L Ataee P Fathollahpour A Lamuch-Deli N. The prevalence of asthma among iranian children and adolescent: a systematic review and meta-analysis. Oxid Med Cell Longev. (2021) 2021:6671870. 10.1155/2021/6671870 34471468 PMC8405330

[19] De Martinis M Sirufo MM Suppa M Di Silvestre D Ginaldi L. Sex and gender aspects for patient stratification in allergy prevention and treatment. Int J Mol Sci. (2020) 21:1535. 10.3390/ijms21041535 32102344 PMC7073150

[20] Siroux V Ballardini N Soler M Lupinek C Boudier A Pin I. The asthma-rhinitis multimorbidity is associated with IgE polysensitization in adolescents and adults. Allergy. (2018) 73:1447–58. 10.1111/all.13410 29331026

